# Bis(dimethyl­ammonium) 2,2′-(1,3,6,8-tetra­oxo-2,7-diaza­pyrene-2,7-di­yl)diacetate

**DOI:** 10.1107/S1600536811026511

**Published:** 2011-07-09

**Authors:** Lan-Ping Xu, Wen-Na Zhao, Lei Han

**Affiliations:** a State Key Laboratory Base of Novel Functional Materials and Preparation Science, Faculty of Materials Science & Chemical Engineering, Ningbo University, Ningbo, Zhejiang 315211, People’s Republic of China; b Key Laboratory for Molecular Design and Nutrition Engineering of Ningbo, Ningbo Institute of Technology, Zhejiang University, Ningbo, Zhejiang 315100, People’s Republic of China

## Abstract

The asymmetric unit of title compound, 2C_2_H_8_N^+^·C_18_H_8_N_2_O_8_^2−^, comprises one crystallographically independent dimethyl­ammonium cation and half of a 2,2′-(1,3,6,8-tetra­oxo-2,7-diaza­pyrene-2,7-di­yl)diacetate dianion. The anion lies on an inversion centre and the two carboxyl­ate groups are in *trans* positions based on the naphthaleneteracarb­oxy­lic diimide group. The crystal packing is stabilized by N—H⋯O hydrogen bonds between cations and anions, as well as by π–π inter­actions between the naph­thaleneteracarb­oxy­lic diimide groups [centroid–centroid distance = 4.812 (3) Å].

## Related literature

For organic supra­molecular solids, see: Pantos *et al.* (2007 ▶). For the prediction of organic crystal structures, see: Pigge (2011 ▶).
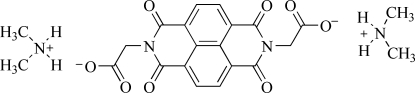


## Experimental

### 

#### Crystal data

2C_2_H_8_N^+^·C_18_H_8_N_2_O_8_^2−^
*M* *_r_* = 472.45
Triclinic, 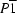

*a* = 4.812 (3) Å
*b* = 8.901 (5) Å
*c* = 12.640 (7) Å
α = 92.361 (9)°
β = 91.512 (6)°
γ = 99.789 (9)°
*V* = 532.7 (5) Å^3^
*Z* = 1
Mo *K*α radiation
μ = 0.11 mm^−1^
*T* = 298 K
0.2 × 0.2 × 0.2 mm

#### Data collection

Rigaku Saturn724+ diffractometer
Absorption correction: multi-scan (*CrystalClear*; Rigaku/MSC, 2008 ▶) *T*_min_ = 0.976, *T*_max_ = 0.983
4207 measured reflections
2298 independent reflections
1969 reflections with *I* > 2σ(*I*)
*R*_int_ = 0.018

#### Refinement

*R*[*F* ^2^ > 2σ(*F* ^2^)] = 0.040
*wR*(*F* ^2^) = 0.114
*S* = 1.04
2298 reflections
202 parameters
All H-atom parameters refined
Δρ_max_ = 0.50 e Å^−3^
Δρ_min_ = −0.23 e Å^−3^

### 

Data collection: *CrystalClear* (Rigaku/MSC, 2008 ▶); cell refinement: *CrystalClear*; data reduction: *CrystalClear*; program(s) used to solve structure: *SHELXS97* (Sheldrick, 2008 ▶); program(s) used to refine structure: *SHELXL97* (Sheldrick, 2008 ▶); molecular graphics: *SHELXTL* (Sheldrick, 2008 ▶); software used to prepare material for publication: *SHELXL97*.

## Supplementary Material

Crystal structure: contains datablock(s) I, global. DOI: 10.1107/S1600536811026511/om2439sup1.cif

Structure factors: contains datablock(s) I. DOI: 10.1107/S1600536811026511/om2439Isup2.hkl

Supplementary material file. DOI: 10.1107/S1600536811026511/om2439Isup3.cml

Additional supplementary materials:  crystallographic information; 3D view; checkCIF report

## Figures and Tables

**Table 1:** Hydrogen-bond geometry (Å, °)

*D*—H⋯*A*	*D*—H	H⋯*A*	*D*⋯*A*	*D*—H⋯*A*
N2—H5⋯O2	1.006 (19)	1.76 (2)	2.7358 (18)	163.5 (16)
N2—H6⋯O1^i^	0.94 (2)	2.13 (2)	2.8419 (18)	131.8 (17)
N2—H6⋯O1^ii^	0.94 (2)	2.13 (2)	2.935 (2)	142.9 (17)

Symmetry codes: (i) 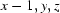; (ii) 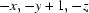.

**Fig. 1.**
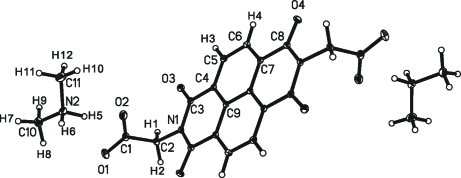
The structure with displacement ellipsoids drawn at the 30% probability level and H atoms shown as small spheres of arbitrary radii.

**Fig. 2.**
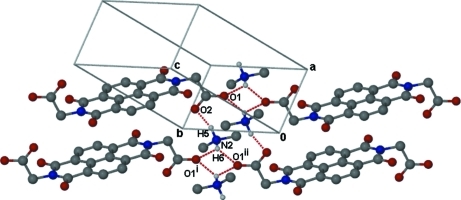
View of N—H···O hydrogen bonds and π···π interactions.

## supplementary crystallographic information

### Comment

The assembly of functionalised organic molecules in the solid state has attracted much attention in crystal engineering and materials science (Pantos *et al.*, 2007). The prediction of organic crystal structures is a central aim of the development of successful synthetic strategies. In general, precise control over solid state assembly processes will facilitate the synthesis of complex functional materials imbued with desirable optical, electronic, magnetic properties starting from carefully chosen yet relatively simple molecular precursors (Pigge, 2011). We are interested in utilizing acid-functionalized naphthalaleneteracarboxylic diimide derivative as starting materials in crystal engineering approaches to a range of functional organic materials. Herein we report an unexpected organic salt compound 2(C_2_H_8_N).(C_18_H_8_N_2_O_8_), (I), which is prepared under solvothermal reaction from 1,4,5,8-naphthalaleneteracarboxylic diimide-N,N'-diacetic acid and 4,4'-bipyridyl in DMF. The dimethylammonium cations come from *in situ* hydrolysis of DMF molecules.

The asymmetric unit of the title compound comprises one crystallographically independent dimethylammonium cation and half of 1,4,5,8-naphthalaleneteracarboxylic diimide-N,N'-diacetate anion. As shown in Figure 1, the anion lies on an inversion centre, and the two carboxylate groups of the anion are in *trans* positions based on naphthalaleneteracarboxylic diimide plane. There are strong N—H···O hydrogen bonds between dimethylammonium cations and the carboxylate groups of anions, which are listed in Table 1. The overall hydrogen bonding interaction makes a 12-atom ring and a 4-atom ring, as shown in Figure 2. On the other hand, because of the large *π*–conjugated skeleton in the naphthalaleneteracarboxylic diimide moiety, the strong intermolecular *π*···*π* interactions are formed with the perpendicular distance between planes of 3.32 Å. Therefore, the crystal packing of I is stabilized both by N—H···O hydrogen bonds and *π*···*π* interactions.

### Experimental

A mixture of 1,4,5,8-naphthalaleneteracarboxylic diimide-N,N'-diacetic acid (38.32 mg), 4,4'-bipyridyl (31.24 mg) in DMF (3 ml) was sealed in a 25 ml Teflon-lined stainless steel reactor and heated at 373 K for 72 h. Single crystals of the title compound were obtained after cooling the solution to room temperature, and washed with DMF. The yield is calculated 60%.

### Refinement

All H atoms were located in difference maps and refined independently with isotropic displacement parameters.

### Crystal data

**Table:**

2C_2_H_8_N^+^·C_18_H_8_N_2_O_8_^2^^−^	*Z* = 1
*M**_r_* = 472.45	*F*(000) = 248
Triclinic, *P*1	*D*_x_ = 1.473 Mg m^−^^3^
Hall symbol: -P 1	Mo *K*α radiation, λ = 0.71073 Å
*a* = 4.812 (3) Å	Cell parameters from 1620 reflections
*b* = 8.901 (5) Å	θ = 2.3–27.5°
*c* = 12.640 (7) Å	µ = 0.11 mm^−^^1^
α = 92.361 (9)°	*T* = 298 K
β = 91.512 (6)°	Prism, colorless
γ = 99.789 (9)°	0.2 × 0.2 × 0.2 mm
*V* = 532.7 (5) Å^3^	

### Data collection

**Table:**

Rigaku Saturn724+ diffractometer	2298 independent reflections
Radiation source: fine-focus sealed tube	1969 reflections with *I* > 2σ(*I*)
Confocal	*R*_int_ = 0.018
Detector resolution: 28.57 pixels mm^-1^	θ_max_ = 27.5°, θ_min_ = 2.9°
ω scans	*h* = −6→6
Absorption correction: multi-scan (*CrystalClear*; Rigaku/MSC, 2008)	*k* = −11→11
*T*_min_ = 0.976, *T*_max_ = 0.983	*l* = −16→16
4207 measured reflections	

### Refinement

**Table:**

Refinement on *F*^2^	Primary atom site location: structure-invariant direct methods
Least-squares matrix: full	Secondary atom site location: difference Fourier map
*R*[*F*^2^ > 2σ(*F*^2^)] = 0.040	Hydrogen site location: inferred from neighbouring sites
*wR*(*F*^2^) = 0.114	All H-atom parameters refined
*S* = 1.04	*w* = 1/[σ^2^(*F*_o_^2^) + (0.069*P*)^2^ + 0.126*P*] where *P* = (*F*_o_^2^ + 2*F*_c_^2^)/3
2298 reflections	(Δ/σ)_max_ < 0.001
202 parameters	Δρ_max_ = 0.50 e Å^−^^3^
0 restraints	Δρ_min_ = −0.23 e Å^−^^3^

### Special details

**Table:**

Geometry. All esds (except the esd in the dihedral angle between two l.s. planes) are estimated using the full covariance matrix. The cell esds are taken into account individually in the estimation of esds in distances, angles and torsion angles; correlations between esds in cell parameters are only used when they are defined by crystal symmetry. An approximate (isotropic) treatment of cell esds is used for estimating esds involving l.s. planes.
Refinement. Refinement of F^2^ against ALL reflections. The weighted R-factor wR and goodness of fit S are based on F^2^, conventional R-factors R are based on F, with F set to zero for negative F^2^. The threshold expression of F^2^ > 2sigma(F^2^) is used only for calculating R-factors(gt) etc. and is not relevant to the choice of reflections for refinement. R-factors based on F^2^ are statistically about twice as large as those based on F, and R- factors based on ALL data will be even larger.

### Fractional atomic coordinates and isotropic or equivalent isotropic displacement parameters (Å^2^)

**Table:**

	*x*	*y*	*z*	*U*_iso_*/*U*_eq_	
O1	0.4577 (2)	0.61234 (11)	0.08100 (7)	0.0248 (3)	
O2	0.1971 (2)	0.79037 (11)	0.12376 (7)	0.0252 (3)	
O3	0.63240 (19)	1.06673 (10)	0.27584 (7)	0.0179 (2)	
O4	−0.2062 (2)	1.40383 (10)	0.63370 (7)	0.0207 (2)	
N1	0.4256 (2)	0.83103 (11)	0.32377 (7)	0.0136 (2)	
N2	−0.0973 (3)	0.68928 (13)	−0.06090 (8)	0.0204 (3)	
H5	0.030 (4)	0.711 (2)	0.0042 (15)	0.038 (5)*	
H6	−0.248 (5)	0.615 (2)	−0.0421 (16)	0.046 (6)*	
C1	0.3916 (3)	0.71603 (14)	0.14037 (9)	0.0180 (3)	
C2	0.5758 (3)	0.75754 (15)	0.24241 (9)	0.0160 (3)	
H1	0.736 (4)	0.827 (2)	0.2294 (13)	0.029 (4)*	
H2	0.634 (3)	0.6701 (19)	0.2725 (12)	0.022 (4)*	
C3	0.4599 (2)	0.99015 (14)	0.32887 (9)	0.0136 (3)	
C4	0.2780 (3)	1.06025 (13)	0.40288 (9)	0.0130 (3)	
C5	0.2994 (3)	1.21692 (14)	0.41186 (9)	0.0151 (3)	
H3	0.429 (4)	1.2802 (18)	0.3699 (13)	0.023 (4)*	
C6	0.1307 (3)	1.28406 (14)	0.48269 (9)	0.0159 (3)	
H4	0.150 (3)	1.3937 (19)	0.4859 (12)	0.023 (4)*	
C7	−0.0576 (3)	1.19431 (13)	0.54381 (9)	0.0131 (3)	
C8	−0.2309 (3)	1.26571 (14)	0.62007 (9)	0.0147 (3)	
C9	0.0849 (2)	0.96643 (13)	0.46443 (8)	0.0121 (3)	
C10	0.0565 (3)	0.62531 (17)	−0.14666 (11)	0.0257 (3)	
H7	−0.077 (4)	0.587 (2)	−0.2030 (15)	0.034 (5)*	
H8	0.156 (4)	0.544 (2)	−0.1197 (13)	0.030 (4)*	
H9	0.190 (4)	0.711 (2)	−0.1699 (15)	0.042 (5)*	
C11	−0.1980 (4)	0.83071 (19)	−0.09026 (12)	0.0301 (3)	
H12	−0.038 (4)	0.910 (2)	−0.1036 (14)	0.033 (5)*	
H11	−0.315 (4)	0.804 (2)	−0.1546 (17)	0.046 (5)*	
H10	−0.309 (4)	0.866 (2)	−0.0336 (16)	0.042 (5)*	

### Atomic displacement parameters (Å^2^)

**Table:**

	*U*^11^	*U*^22^	*U*^33^	*U*^12^	*U*^13^	*U*^23^
O1	0.0250 (5)	0.0264 (5)	0.0221 (5)	0.0038 (4)	0.0058 (4)	−0.0082 (4)
O2	0.0258 (5)	0.0310 (6)	0.0201 (5)	0.0102 (4)	−0.0030 (4)	−0.0031 (4)
O3	0.0183 (5)	0.0194 (5)	0.0164 (4)	0.0034 (4)	0.0055 (3)	0.0028 (3)
O4	0.0297 (5)	0.0129 (4)	0.0205 (4)	0.0063 (4)	0.0062 (4)	−0.0011 (3)
N1	0.0163 (5)	0.0152 (5)	0.0102 (5)	0.0054 (4)	0.0016 (4)	−0.0011 (4)
N2	0.0210 (6)	0.0233 (6)	0.0165 (5)	0.0028 (5)	−0.0013 (4)	0.0024 (4)
C1	0.0181 (6)	0.0202 (6)	0.0150 (6)	0.0012 (5)	0.0045 (5)	−0.0003 (4)
C2	0.0177 (6)	0.0174 (6)	0.0144 (5)	0.0071 (5)	0.0042 (5)	−0.0015 (4)
C3	0.0144 (6)	0.0165 (6)	0.0103 (5)	0.0043 (5)	−0.0016 (4)	0.0008 (4)
C4	0.0144 (6)	0.0155 (6)	0.0098 (5)	0.0044 (5)	−0.0002 (4)	0.0005 (4)
C5	0.0161 (6)	0.0153 (6)	0.0137 (5)	0.0018 (5)	0.0018 (4)	0.0028 (4)
C6	0.0202 (6)	0.0120 (6)	0.0158 (6)	0.0039 (5)	0.0004 (5)	0.0004 (4)
C7	0.0153 (6)	0.0139 (6)	0.0108 (5)	0.0045 (5)	−0.0005 (4)	0.0000 (4)
C8	0.0173 (6)	0.0153 (6)	0.0122 (5)	0.0052 (5)	−0.0006 (4)	0.0008 (4)
C9	0.0134 (6)	0.0130 (6)	0.0101 (5)	0.0033 (5)	−0.0015 (4)	0.0005 (4)
C10	0.0313 (8)	0.0248 (7)	0.0201 (6)	0.0023 (7)	0.0041 (6)	0.0003 (5)
C11	0.0319 (8)	0.0319 (8)	0.0293 (7)	0.0117 (7)	0.0000 (6)	0.0078 (6)

### Geometric parameters (Å, °)

**Table:**

O1—C1	1.2535 (16)	C4—C9	1.4119 (18)
O2—C1	1.2529 (17)	C5—C6	1.4061 (17)
O3—C3	1.2156 (15)	C5—H3	0.954 (18)
O4—C8	1.2195 (16)	C6—C7	1.3775 (19)
N1—C8^i^	1.3909 (17)	C6—H4	0.963 (16)
N1—C3	1.3963 (17)	C7—C9^i^	1.4135 (18)
N1—C2	1.4669 (15)	C7—C8	1.4834 (16)
N2—C10	1.4771 (18)	C8—N1^i^	1.3909 (17)
N2—C11	1.4811 (19)	C9—C9^i^	1.412 (2)
N2—H5	1.006 (19)	C9—C7^i^	1.4135 (18)
N2—H6	0.94 (2)	C10—H7	0.958 (19)
C1—C2	1.5410 (18)	C10—H8	0.999 (18)
C2—H1	0.924 (18)	C10—H9	0.97 (2)
C2—H2	0.961 (16)	C11—H12	0.977 (19)
C3—C4	1.4872 (16)	C11—H11	0.98 (2)
C4—C5	1.3804 (19)	C11—H10	0.98 (2)
			
C8^i^—N1—C3	125.18 (10)	C6—C5—H3	119.6 (9)
C8^i^—N1—C2	116.13 (10)	C7—C6—C5	120.39 (12)
C3—N1—C2	118.08 (10)	C7—C6—H4	121.9 (9)
C10—N2—C11	112.51 (11)	C5—C6—H4	117.7 (9)
C10—N2—H5	108.9 (11)	C6—C7—C9^i^	120.41 (11)
C11—N2—H5	109.7 (10)	C6—C7—C8	120.22 (11)
C10—N2—H6	109.1 (13)	C9^i^—C7—C8	119.36 (11)
C11—N2—H6	110.9 (12)	O4—C8—N1^i^	120.54 (11)
H5—N2—H6	105.5 (16)	O4—C8—C7	121.96 (12)
O2—C1—O1	126.66 (12)	N1^i^—C8—C7	117.50 (11)
O2—C1—C2	117.51 (11)	C4—C9—C9^i^	119.72 (14)
O1—C1—C2	115.81 (11)	C4—C9—C7^i^	121.20 (11)
N1—C2—C1	111.40 (10)	C9^i^—C9—C7^i^	119.08 (14)
N1—C2—H1	106.2 (10)	N2—C10—H7	107.9 (11)
C1—C2—H1	110.7 (11)	N2—C10—H8	110.6 (10)
N1—C2—H2	107.6 (10)	H7—C10—H8	111.8 (14)
C1—C2—H2	112.6 (10)	N2—C10—H9	105.1 (11)
H1—C2—H2	108.1 (14)	H7—C10—H9	110.1 (15)
O3—C3—N1	121.18 (11)	H8—C10—H9	111.0 (14)
O3—C3—C4	122.08 (11)	N2—C11—H12	110.3 (11)
N1—C3—C4	116.74 (11)	N2—C11—H11	106.1 (12)
C5—C4—C9	120.10 (11)	H12—C11—H11	110.3 (15)
C5—C4—C3	119.99 (11)	N2—C11—H10	110.0 (11)
C9—C4—C3	119.92 (11)	H12—C11—H10	109.6 (15)
C4—C5—C6	120.29 (12)	H11—C11—H10	110.4 (16)
C4—C5—H3	120.1 (9)		
			
C8^i^—N1—C2—C1	79.11 (13)	C3—C4—C5—C6	−179.17 (10)
C3—N1—C2—C1	−92.46 (13)	C4—C5—C6—C7	0.01 (19)
O2—C1—C2—N1	23.91 (16)	C5—C6—C7—C9^i^	−0.44 (19)
O1—C1—C2—N1	−157.63 (11)	C5—C6—C7—C8	178.71 (10)
C8^i^—N1—C3—O3	−178.82 (10)	C6—C7—C8—O4	−2.53 (19)
C2—N1—C3—O3	−8.08 (17)	C9^i^—C7—C8—O4	176.63 (11)
C8^i^—N1—C3—C4	1.85 (17)	C6—C7—C8—N1^i^	177.33 (10)
C2—N1—C3—C4	172.59 (9)	C9^i^—C7—C8—N1^i^	−3.50 (17)
O3—C3—C4—C5	0.90 (18)	C5—C4—C9—C9^i^	−0.3 (2)
N1—C3—C4—C5	−179.78 (10)	C3—C4—C9—C9^i^	179.21 (12)
O3—C3—C4—C9	−178.65 (10)	C5—C4—C9—C7^i^	179.58 (10)
N1—C3—C4—C9	0.67 (17)	C3—C4—C9—C7^i^	−0.87 (18)
C9—C4—C5—C6	0.38 (19)		

Symmetry codes: (i) −*x*, −*y*+2, −*z*+1.

### Hydrogen-bond geometry (Å, °)

**Table:**

*D*—H···*A*	*D*—H	H···*A*	*D*···*A*	*D*—H···*A*
N2—H5···O2	1.006 (19)	1.76 (2)	2.7358 (18)	163.5 (16)
N2—H6···O1^ii^	0.94 (2)	2.13 (2)	2.8419 (18)	131.8 (17)
N2—H6···O1^iii^	0.94 (2)	2.13 (2)	2.935 (2)	142.9 (17)

Symmetry codes: (ii) *x*−1, *y*, *z*; (iii) −*x*, −*y*+1, −*z*.
